# Prosthesis-patient mismatch following transcatheter aortic valve replacement for degenerated transcatheter aortic valves: the TRANSIT-PPM international project

**DOI:** 10.3389/fcvm.2022.931207

**Published:** 2022-07-29

**Authors:** Luca Testa, Matteo Casenghi, Enrico Criscione, Nicolas M. Van Mieghem, Didier Tchétché, Anita W. Asgar, Ole De Backer, Azeem Latib, Bernhard Reimers, Giulio Stefanini, Carlo Trani, Francesco Giannini, Antonio Bartorelli, Wojtek Wojakowski, Maciej Dabrowski, Dariusz Jagielak, Adrian P. Banning, Rajesh Kharbanda, Raul Moreno, Joachim Schofer, Christina Brinkmann, Niels van Royen, Duane Pinto, Antoni Serra, Amit Segev, Arturo Giordano, Nedy Brambilla, Mauro Agnifili, Antonio Popolo Rubbio, Mattia Squillace, Jacopo Oreglia, Rudolph Tanja, James M. McCabe, Alexander Abizaid, Michiel Voskuil, Rui Teles, Giuseppe Biondi Zoccai, Lars Sondergaard, Francesco Bedogni

**Affiliations:** ^1^IRCCS Policlinico S. Donato, Milan, Italy; ^2^Erasmus University Medical Center, Rotterdam, Netherlands; ^3^Groupe CardioVasculaire Interventionnel, Clinique Pasteur, Toulouse, France; ^4^Montreal Heart Institute, Montreal, QC, Canada; ^5^Rigshospitalet, Copenhagen University Hospital, Copenhagen, Denmark; ^6^Montefiore Medical Center, New York, NY, United States; ^7^CCS Humanitas Research Hospital, Rozzano-Milan, Italy; ^8^Department of Biomedical Sciences, Humanitas University, Pieve Emanuele-Milan, Italy; ^9^Policlinico Universitario A. Gemelli, Rome, Italy; ^10^Maria Cecilia Hospital, Cotignola, Ravenna, Italy; ^11^Centro Cardiologico Monzino, IRCCS, Milan, Italy; ^12^Department of Biomedical and Clinical Sciences “Luigi Sacco”, University of Milan, Milan, Italy; ^13^Medical University of Silesia, Katowice, Poland; ^14^Department of Interventional Cardiology and Angiology, National Institute of Cardiology, Warsaw, Poland; ^15^Department of Cardiology, Medical University, Gdańsk, Poland; ^16^John Radcliffe Hospital, Oxford, United Kingdom; ^17^Hospital La Paz, IdiPAZ, CIBER-CV, Madrid, Spain; ^18^MVZ Department Structural Heart Disease at St. Georg, Hamburg, Germany; ^19^Radboud University Medical Center, Nijmegen, Netherlands; ^20^Beth Israel Deaconess Medical Center, Boston, MA, United States; ^21^Hospital de la Santa Creu i Sant Pau, Barcelona, Spain; ^22^The Heart and Vascular Center, Chaim Sheba Medical Center, Ramat Gan, Israel; ^23^Pineta Grande Hospital, Caserta, Italy; ^24^Niguarda Ca Granda Hospital, Milan, Italy; ^25^Heart and Diabetes Center NRW, Bad Oeynhausen, Germany; ^26^University of Washington, Seattle, WA, United States; ^27^Instituto do Coração (Incor), São Paulo, Brazil; ^28^University Medical Center, Utrecht, Netherlands; ^29^Hospital de Santa Cruz, Centro Hospitalar de Lisboa Ocidental, Lisbon, Portugal; ^30^Department of Medical-Surgical Sciences and Biotechnologies, Sapienza University of Rome, Latina, Italy; ^31^Mediterranea Cardiocentro, Naples, Italy

**Keywords:** TAVR, failed TAVR, TAVR in TAVR, prosthesis-patient mismatch, mortality

## Abstract

**Background:**

A severe prosthesis-patient mismatch (PPM) is associated with adverse outcomes following transcatheter aortic valve replacement (TAVR) for *de novo* aortic stenosis or a failed surgical bioprosthesis. The impact of severe PPM in patients undergoing TAV-in-TAVR is unknown.

**Aim:**

We sought to investigate the incidence and 1-year outcomes of different grades of PPM in patients undergoing TAV-in-TAVR.

**Materials and methods:**

The TRANSIT-PPM is an international registry, including cases of degenerated TAVR treated with a second TAVR. PPM severity, as well as in-hospital, 30-day, and 1-year outcomes were defined according to the Valve Academic Research Consortium-3 (VARC-3) criteria.

**Results:**

Among 28 centers, 155 patients were included. Severe PPM was found in 6.5% of patients, whereas moderate PPM was found in 14.2% of patients. The rate of severe PPM was higher in patients who underwent TAV-in-TAVR with a second supra-annular self-expanding (S-SE) TAVR (10%, *p* = 0.04). Specifically, the rate of severe PPM was significantly higher among cases of a SE TAVR implanted into a balloon-expandable (BE) device (19%, *p* = 0.003). At 1-year follow-up, the rate of all-cause mortality, and the rate of patients in the New York Heart Association (NYHA) class III/IV were significantly higher in the cohort of patients with severe PPM (*p* = 0.016 and *p* = 0.0001, respectively). Almost all the patients with a severe PPM after the first TAVR had a failed < 23 mm BE transcatheter heart valve (THV): the treatment with an S-SE resolved the severe PPM in the majority of the cases.

**Conclusion:**

After TAV-in-TAVR, in a fifth of the cases, a moderate or severe PPM occurred. A severe PPM is associated with an increased 1-year all-cause mortality.

**Clinical trial registration:**

[https://clinicaltrials.gov], identifier [NCT04500964].

## Introduction

Prosthesis-patient mismatch (PPM) may occur after surgical aortic valve replacement (SAVR) or transcatheter aortic valve replacement (TAVR) when a normally functioning prosthetic valve presents an effective orifice area (EOA) relatively small for the patient’s body surface area (BSA), thus not allowing an adequate cardiac output (1). Several studies on patients undergoing SAVR showed that severe PPM was associated with increased mortality and structural valve degeneration, regardless of its severity, in the postoperative period (2, 3). On the other hand, patients treated by means of transcatheter valves, which are characterized by a larger EOA and lower gradient compared to surgical valves, experience a lower incidence of severe PPM: the clinical impact of severe PPM is still controversial (4, 5). Recently, TAVR for a failed surgical bioprosthetic aortic valve [TAVR-valve-in-valve (ViV)] has emerged as an attractive option for patients who are at an increased risk for a surgical redo; although, according to a recent meta-analysis, it may be associated with a higher incidence of severe PPM as compared to redo-SAVR (6, 7). Indeed, over 30% of TAVR-ViV procedures in the Society of Thoracic Surgeons (STS)/the American College of Cardiology, the Transcatheter Valve Therapy (TVT), and the Valve-in-Valve International Database (VIVID) Registries resulted in an elevated postprocedural transvalvular gradient (4, 6). Although, rarely, transcatheter aortic valves can also degenerate (8): the TRANSIT international project collected the largest series of patients with a degenerated TAVR treated by means of a second TAVR (TAV-in-TAVR) and, consistently with a previous smaller registry, showed acceptable procedural and 1-year outcomes (9, 10). In the present TRANSIT-PPM study, we sought to evaluate the incidence and impact of severe PPM on outcomes, in patients undergoing TAV-in-TAVR.

## Materials and methods

The TRANSIT-PPM project is an investigator-initiated international multicenter registry, including consecutive patients undergoing TAVR for a degenerated transcatheter aortic valve (ClinicalTrials.gov Identifier: NCT04500964). We evaluated cases performed with supra-annular self-expanding (S-SE) (CoreValve, Evolut R, and Evolut PRO) and intra-annular balloon-expandable (BE) transcatheter heart valves (THVs) (Edwards SAPIEN, SAPIEN XT, and SAPIEN S3).

Data concerning procedural results and echocardiographic parameters after each TAVR were collected. Data concerning the last available follow-up were also collected. This study was approved by an institutional review committee and the subjects gave informed consent.

## Definitions

The registry exclusively collected cases of degenerated TAV treated by means of a second TAVR. Patients undergoing TAV-in-TAVR due to a procedural failure of the indexed TAVR were not included.

Procedural, device success, as well as PPM were defined according to the Valve Academic Research Consortium-3 (VARC-3) definitions (11). In particular, PPM was defined moderate if the predicted EOA was > 0.65 and < 0.85 cm^2^/m^2^ for patients with body mass index (BMI) < 30 kg/m^2^, or > 0.55 and < 0.70 cm^2^/m^2^ for patients with BMI > 30 kg/m^2^, and severe if the predicted EOA was ≤ 0.65 cm^2^/m^2^ for patients with BMI < 30 kg/m^2^ and ≤ 0.55 for patients with BMI > 30 kg/m^2^ (8–10).

The left ventricular outflow tract (LVOT) measures have been obtained with the CT scan that all the patients performed before the procedure.

## Statistical analysis

Descriptive statistics are reported as mean and SD for normally distributed continuous variables, as median and 25–75th percentile otherwise. Absolute and relative frequencies are reported for categorical variables. For continuous variables, the comparisons were done either with ANOVA or with a non-parametric test (Kruskal–Wallis test). For categorical variables, comparisons among groups were done with the chi-squared tests or Fisher’s exact tests. All-cause death was reported using the Kaplan–Meier estimates together with their 95% CI. The Wilcoxon signed rank sum test was used for the comparison of echo parameters in paired analyzes. The cumulative incidences of clinical events at follow-up were assessed with the Kaplan–Meier method and log-rank test. A two-sided *P*-value of < 0.05 was considered statistically significant. Statistical analysis was performed using SPSS software version 23 (IBM Incorporation, Armonk, NY, United States).

## Results

Because of the sensitive nature of the data collected for this study, requests to access the dataset from qualified researchers trained in human subject confidentiality protocols may be sent to the corresponding author.

The TRANSIT project is an investigator-initiated registry that started collecting data in January 2020 (ClinicalTrials.gov Identifier: NCT04500964). A group of 28 centers took part in the project: 22 in Europe, 4 in North America, 1 in South America, and 1 in the Middle East. Among a total number of about 40,000 procedures performed since 2008, 155 cases of TAV-in-TAVR were eventually included in the TRANSIT-PPM study. Of these, 73 (47%) cases presented a degenerated supra-annular self-expanding valve, while 82 (53%) cases had a degenerated balloon-expandable device.

According to the VARC-3 definitions, 8 (5.2%) and 32 (20.6%) patients, respectively, presented a severe or moderate PPM after the first procedure, while no patients had a mean residual gradient higher than 20 mm Hg or a more than mild aortic regurgitation (AR).

The mean age was 77.9 ± 7.7 years and the male gender was slightly more represented (57.4%). The majority of patients (74%) were in the NYHA class III or IV at admission. The mean left ventricular ejection fraction was 49 ± 13.4. The European System for Cardiac Operative Risk Evaluation I (EuroSCORE I) was 20.3 ± 15.0, the EuroSCORE II was 8.7 ± 7.5, and the STS score was 6.3 ± 5 (Table 1). Most patients (57%) had a mainly regurgitant degenerated bioprosthesis, 52 (34%) patients had a stenotic degenerated THV, and 15 (10%) patients had a mixed degeneration of the first implanted valve (Table 1).

**TABLE 1:** Demographic characteristics of the study population.

	Overall (*N* = 155)	Severe PPM (*N* = 10)	Moderate PPM (*N* = 22)	None (*N* = 123)	*P*-value
Age	77.9 ± 7.7	77.5 ± 7.6	77.9 ± 7.5	79.2 ± 8.7	0.2
Male	89 (57.4%)	2 (20%)	8 (36%)	79 (64%)	0.002
BSA (m2)	1.8 ± 0.2	1.7 ± 0.07	1.8 ± 0.2	1.8 ± 0.2	0.4
BMI < 21 kg/m2	25 (16%)	1 (10%)	2 (9%)	22 (18%)	0.5
BMI > 30 kg/m2	19 (12%)	0 (0)	2 (9%)	17 (14%)	0.4
Hypertension	135 (87%)	10 (100%)	22 (100%)	103 (84%)	0.05
Dyslipidemia	104 (67%)	6 (60%)	20 (91%)	78 (65%)	0.04
Diabetes	19 (12%)	4 (40%)	6 (27%)	32 (26%)	0.7
Smoker	40 (26%)	2 (20%)	4 (21%)	34 (34%)	0.4
COPD	36 (23%)	3 (30%)	6 (27%)	27 (22%)	0.8
Severe renal failure	28 (18%)	3 (30%)	2 (9%)	23 (19%)	0.3
Dialysis	6 (4%)	1 (10%)	0 (0)	5 (4%)	0.4
Stroke	9 (6%)	1 (10%)	0 (0)	8 (7%)	0.4
Previous pacemaker	50 (32%)	3 (30%)	6 (27%)	41 (34%)	0.8
Previous cardiac surgery	30 (19%)	3 (30%)	4 (18%)	23 (19%)	0.3
HISTORY of MI	42 (27%)	2 (20%)	8 (36%)	32 (26%)	0.5
Previous PCI	65 (42%)	5 (50%)	6 (27%)	54 (45%)	0.3
NYHA III/IV	114 (74%)	7 (70%)	18 (82%)	89 (74%)	0.7
LV ejection Fraction (%)	49 ± 13.4	52.5 ± 10.3	52.6 ± 18.7	48.1 ± 13.1	0.3
Aortic valve area (cm^2^)	1.34 ± 0.7	0.75 ± 0.3	1.05 ± 0.5	1.44 ± 0.72	0.03
Euroscore I	20.3 ± 15.0	37.4 ± 17.3	18.4 ± 7.3	18.1 ± 13.0	0.01
Euroscore II	8.7 ± 7.5	15.5 ± 15.0	9.5 ± 1.8	8.2 ± 7.6	0.06
STS Score	6.3 ± 5.9	12.3 ± 14.9	4.7 ± 1.2	6.9 ± 5.7	0.03
Regurgitant degenerated	88 (57%)	3 (30%)	10 (46%)	75 (61%)	0.08
Stenotic degenerated	52 (34%)	3 (20%)	12 (55%)	37 (30%)	0.09
Mixed degenerated	15 (10%)	4 (40%)	0	11 (9%)	0.001

BSA, body surface area; BMI, body mass index; COPD, chronic obstructive pulmonary disease; LV, left ventricle; MI, myocardial infarction; NYHA, New York Heart Association; PCI, percutaneous coronary intervention; STS, Society of Thoracic Surgeons.

Patients were grouped and analyzed according to the grade of PPM after the second TAVR: 10 (6.5%) patients had severe PPM, 22 (14.2%) patients had moderate PPM, and 123 (79.3%) patients had no PPM.

There were no differences in BSA and BMI distribution between the groups (Table 1). Overall, patients were frequently hypertensive (87%) and dyslipidemic (64%); in particular, the rate of the aforementioned risk factor was higher in patients with moderate or severe PPM (*p* = 0.05 and *p* = 0.04, respectively). No other differences were found among common risk factors such as diabetes, chronic obstructive pulmonary disease (COPD), and severe renal failure (Table 1). Risk scores (EuroSCORE I, EuroSCORE II, and STS), as well as mean postprocedural transvalvular gradient, were significantly higher in patients with severe PPM compared to those with moderate or none/mold PPM (*p* = 0.03 and *p* = 0.01, respectively).

Of note, 4 out of 10 patients presenting a severe PPM after TAV-in-TAVR belong to the mixed-degenerated cohort (*p* = 0.001).

## Assessment of the prosthesis-patient mismatch before transcatheter aortic valve-in-transcatheter aortic valve replacement

All the cases of severe PPM after the first TAVR concerned patients with a BE THV (8 patients), with a significantly higher prevalence of ≤ 23 mm THVs (7 out of 8); conversely, no grade of PPM was more frequent among patients with an S-SE THV, in particular in patients with a > 23 mm THV (Supplementary Tables 1, 2).

## Procedural results

In this cohort of patients with a degenerated first THV undergoing TAV-in-TAVR, an S-SE THV was implanted in 86 cases (55%), while a BE THV was implanted in the remaining 69 cases (45%) (see Table 2 for the procedural results). Supplementary Table 3 shows the iterations of the first and second THV according to the size ≥ 23 mm.

**TABLE 2:** Procedural data.

Variables	Overall (*N* = 155)	Severe (*N* = 10)	Moderate (*N* = 22)	None (*N* = 123)	*P*-value
Transfemoral approach	141 (91%)	10 (100%)	18 (81,8%)	113 (91,9%)	0,19
Predilatation	28 (18,1%)	3 (30%)	4 (18,2%)	21 (17,1%)	0,59
Postdilatation	63 (40,6%)	5 (50%)	8 (36,4%)	50	0,77
Contrast (mean ± SD)	93 (18)	93,6 (20)	82,7 (34)	94 (24)	0.3
Aortic dissection	0	0	0	0	–
Annulus rupture	1 (0,6%)	0	0	1 (0,8%)	0,88
Valve embolization	0	0	0	0	–
Myocardial infarction	1 (0,6%)	0	0	1 (0,8%)	0,88
Emergency surgery	1 (0,6%)	0	0	1 (0,8%)	0,88
Coronary obstruction	0	0	0	0	–
Stroke/TIA	0	0	0	0	–
Cardiac tamponade	0	0	0	0	–
Major vascular complication	3 (1,9%)	0	0	3 (2,4%)	0,67
Ventricular arrhythmias	0	0	0	0	–
Device success	126 (81,3%)	2 (20%)	16 (72,7%)	108 (87,8%)	0,12
Mean gradient (mmHg, mean ± SD)	10,3 (4)	11,9 (5)	8,8 (7)	10,4 (4)	0.15

AR, aortic regurgitation; TIA, transient ischemic attack.

We could not find a specific strategy in the selection of the second TAVR except at the operator’s discretion.

**FIGURE 1:**
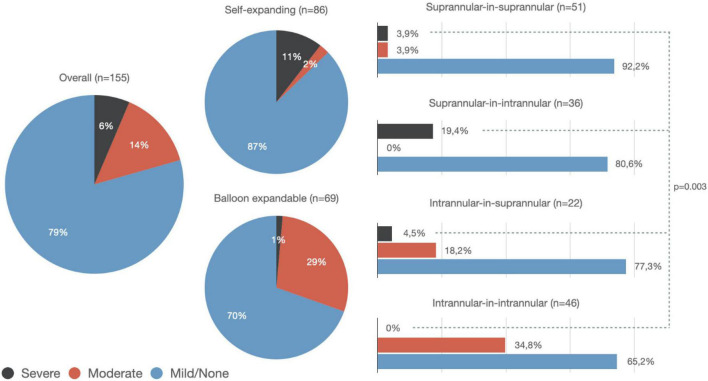
Rate of prosthesis-patient mismatch among the overall population (left-sided), patients treated with a second self-expanding transcatheter heart valve (THV) (top center), and patients treated with a second balloon-expandable THV (bottom center). Incidence of severe prosthesis-patient mismatch (PPM) was higher in patients with supra-annular-in-intra-annular THV (*p* = 0.003). Particularly, a higher rate of severe PPM was observed among the supra-annular-in-intra-annular group compared to the supra-annular-in-supra-annular or intra-annular-in-intra-annular groups (*p* = 0.02 and *p* = 0.002, respectively).

The cohort of patients treated by means of an S-SE showed a significantly higher rate of severe PPM compared to those who received a BE (10.4 vs. 1.5%, *p* = 0.04) (Figure 1). On the contrary, the rate of moderate PPM was significantly higher in those patients receiving a BE THV (2.3 vs. 29%, *p* = 0.0001).

More in detail, the rate of severe PPM was significantly higher in those patients who received an S-SE device to treat a degenerated BE THV (7/10, *p* = 0.003). The rate of moderate PPM was significantly higher when a BE THV has been used to treat a degenerated BE THV (16/22, *p* = 0.0001) (Table 3 and Figure 1).

**TABLE 3:** First-second transcatheter aortic valve replacement (TAVR) combinations and subsequent grades of prosthesis-patient mismatch (PPM) (see text for acronyms).

	Overall (*N* = 155)	Severe PPM (*N* = 10)	Moderate PPM (*N* = 22)	None (*N* = 123)	*P*-value
2nd S-SE	86 (55%)	9 (90%)	2 (9%)	75 (61%)	0.0001
2nd BE	69 (45%)	1 (10%)	20 (91%)	48 (39%)	0.0001
S-Se in S-SE	51 (33%)	2 (20%)	2 (9%)	47 (38%)	0.02
S-Se in BE	36 (23%)	7 (70%)	0 (0)	29 (24%)	0.0001
BE in S-Se	22 (14%)	1 (10%)	4 (18%)	17 (14%)	0.8
BE in BE	46 (29%)	0 (0)	16 (73%)	30 (24%)	0.0001

Overall, the rates of severe and moderate PPM were significantly higher in patients presenting with a degenerated ≤ 23 mm THV (Table 4): in particular, 9 out of 10 cases of severe PPM after the second TAVR occurred in patients with a degenerated first THV of ≤ 23 mm in size.

**TABLE 4:** Analysis of the prosthesis-patient mismatch (PPM) occurrence after the second transcatheter aortic valve replacement (TAVR), according to the size of the first transcatheter heart valve (THV).

	Overall (*N* = 155)	1st TAVR ≤ 23 mm (*N* = 55)	1st TAVR > 23 mm (*N* = 100)	*P*-value
Severe PPM	10 (6.5%)	9 (16.4%)	1 (1%)	0.001
Moderate PPM	22 (20.6%)	14 (25.5%)	8 (8%)	0.004
No PPM	123 (74.2%)	32 (58.2%)	91 (91%)	0.0001

All the patients with a severe PPM after the first TAVR have been treated with an S-SE: only 1 out of 8 patients had a severe PPM after the second TAVR (Table 5).

**TABLE 5:** Different grades of prosthesis-patient mismatch (PPM) after TAV-in- transcatheter aortic valve replacement (TAVR) according to the PPM of the degenerated TAVR.

	Overall (*N* = 155)	Severe PPM 2nd TAVR (*N* = 10)	Moderate PPM 2nd TAVR (*N* = 22)	None (N = 123) 2nd TAVR (*N* = 123)	*P*-value
Severe PPM 1st TAVR	8 (5.2%)	1 (10%)	2 (9.1%)	5 (4.1%)	0.5
Moderate PPM 1st TAVR	32 (20.6%)	6 (60%)	8 (36.4%)	18 (14.6%)	0.0001
No PPM 1st TAVR	115 (74.2%)	3 (30%)	12 (54.5%)	100 (81.3%)	0.0001

The VARC-3 defined procedural success rate was 80.6% with 22 (14.2%) patients presenting: a severe PPM (9 patients) and/or residual gradient ≥ 20 mm Hg (13 patients), and 2 (1.3%) patients showing a more than mild AR.

The presence of a no PPM after the first TAVR [hazard ratio (HR) 0.126, 0.31–0.51, *p* = 0.004], and of a degenerated THV of ≤ 23 mm (HR 19.7, 2.28–157.4, *p* = 0.006) were independent predictors of severe PPM after the second TAVR.

## In-hospital outcomes

Seven patients (4.5%) died during the in-hospital stay, all due to cardiovascular (CV) causes. None presented a severe PPM, while 4 patients had moderate PPM and 3 patients had no significant PPM (*p* = 0.006). Two patients had a myocardial infarction during the hospital stay. No differences in the incidence of conduction disturbances, pacemaker (PM) implantation, or new-onset atrial fibrillation were observed according to the presence and severity of PPM. Other in-hospital outcomes are shown in Table 6.

**TABLE 6:** In-hospital outcomes.

	Overall (*N* = 155)	Severe PPM (*N* = 10)	Moderate PPM (*N* = 22)	None (*N* = 123)	*P*-value
All cause mortality	7 (4.5%)	0 (0)	4 (18%)	3 (2%)	0.003
Cardiovascular mortality	7 (4.5%)	0 (0)	4 (18%)	3 (2%)	0.006
New onset LBBB	2 (1.3%)	0 (0)	0 (0)	2 (1.6%)	0.8
New onset AF	6 (4%)	1 (10%)	0 (0)	5 (4%)	0.4
New PM	6 (4%)	0 (0)	0 (0)	6 (5%)	0.5
Stroke/TIA	6 (4%)	0 (0)	0 (0)	6 (5%)	0.5
Major vascular complications	4 (2.6%)	0 (0)	2 (9%)	2 (1.6%)	0.1
Major bleeding (≥ BARC-3a)	9 (6%)	1 (10%)	0 (0)	8 (7%)	0.4
MI	2 (1.3%)	1 (10%)	0 (0)	1 (0.8%)	0.04
Valve thrombosis	0 (0)	–	–	–	–
AKI (≥ AKIN-2)	7 (4.5%)	1 (10%)	2 (9%)	4 (3%)	0.3
Sepsis	8 (5%)	1 (10%)	2 (9%)	5 (4%)	0.4

LBBB, left bundle branch block; AF, atrial fibrillation; PM, pacemaker; TIA, transient ischemic attack; BARC, Bleeding Academic Research Consortium; MI, myocardial infarction; AKI, acute kidney injury; AKIN, acute kidney injury network classification.

## 30-day and 1-year follow-up

A 30-day cumulative overall mortality rate was 7.1% with no further cardiovascular death and no significant differences reported among groups (*p* = 0.08). Compared to patients with moderate or no PPM, those patients with a severe PPM showed a higher rate of valve-related hospitalization (*p* = 0.001) and dyspnea at rest or on mild exertion (the NYHA class III/IV) (*p* = 0.001) (Table 7). Two cases of valve thrombosis had been detected, both in patients with moderate PPM (*p* = 0.001).

**TABLE 7:** Cumulative 30-day and 1-year outcomes.

	Overall (*N* = 155)	Severe PPM (*N* = 10)	Moderate PPM (*N* = 22)	None (*N* = 123)	*P*-value
30-day					
CV death	7 (4.5%)	0 (0)	4 (18%)	3 (2.4%)	0.003
All-cause death	11 (7.1%)	1 (10%)	4 (18%)	6 (3.8%)	0.08
Valve related hospitalization	5 (3%)	2 (20%)	0 (0)	3 (2.4%)	0.001
Valve thrombosis	2 (1.3%)	0 (0)	2 (9%)	0 (0)	0.001
NYHA class III-IV	12 (7.7%)	3 (30%)	0 (0)	9 (7.4%)	0.0001
1-year					
All-cause death	20 (12.9%)	4 (40%)	4 (18%)	12 (9.7%)	0.016
CV death	9 (5.8%)	2 (20%)	4 (18%)	3 (2.4%)	0.002
Valve related hospitalization	10 (6.5%)	2 (20%)	2 (9%)	6 (5%)	0.15
Valve thrombosis	2 (1.3%)	0 (0)	2 (9%)	0 (0)	0.002
NYHA class III-IV	9 (5.8%)	2 (20%)	0 (0)	7 (5.7%)	0.0001

CV, cardiovascular.

Cumulative 1-year all-cause mortality was 12.9% (a miscellaneous of pneumonia, sepsis, CV death, and cancer) with a CV-related death occurring in 5.8% of patients. Compared to patients with no and moderate PPM, the rate of all-cause mortality was significantly higher in patients with a severe mismatch (*p* = 0.016).

**FIGURE 2:**
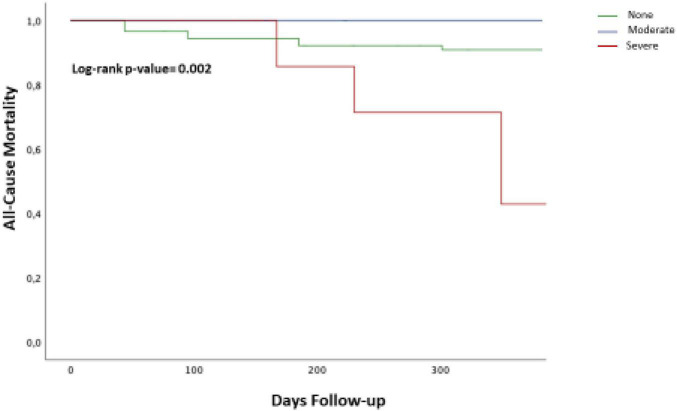
The Kaplan–Meier curves of cumulative 1-year all-cause death according to the presence of severe prosthesis-patient mismatch (PPM). The cumulative all-cause mortality rate at 1 year in patients with a severe was higher as compared with patients with moderate PPM or no PPM (log-rank *p*-value = 0.001). Blue line = Moderate/none PPM; Red line = Severe PPM.

With respect to patients with no PPM, both the patients with moderate and severe PPM had a significantly higher rate of cardiac death (*p* = 0.002) (Table 7 and Figure 2).

Valve-related hospitalization occurred in 10 (6.5%) patients, with no significant differences between the groups. The rate of patients in the NYHA class III/IV was significantly higher in the severe PPM cohort (*p* = 0.0001). No cases of valve thrombosis, myocardial infarction (MI), stroke, or valve dysfunction requiring intervention were further recorded.

No differences in the rate of all-cause mortality, CV mortality, and valve-related hospitalization were found among those patients with elevated postprocedural mean gradient (≥ 20 mm Hg), but without severe PPM (*p* = 0.2, *p* = 0.5, and *p* = 0.8, respectively).

## Discussion

A second TAVR to treat a degenerated TAVR is a reasonable option with acceptable in-hospital and 1-year outcomes (9). However, likewise, in the field of TAVR in SAVR, a high residual gradient may occur possibly affecting the clinical outcome, especially when associated with a severe PPM.

The TRANSIT-PPM project is the first multicenter, international registry that evaluated the incidence and clinical outcomes of the different grades of PPM after TAV-in-TAVR.

The main results of our study may be summarized as follows:

• Severe and moderate PPM was found in 6.5 and 14.2% of patients undergoing TAV-in-TAVR, respectively.
• The rate of severe PPM after TAV-in-TAVR was significantly higher in patients treated with an S-SE THV (10.4%, *p* = 0.04), particularly in those with an S-SE THV implanted into a degenerated BE THV (*p* = 0.003).
• The rate of moderate PPM after TAV-in-TAVR was significantly higher in patients treated with a BE THV (2.3 vs. 29%, *p* = 0.0001), particularly in those with a BE THV implanted into a degenerated BE THV.
• A severe PPM after TAV-in-TAVR is significantly more frequent when treating a degenerated ≤ 23 mm THV.
• In the majority of the cases of a degenerated BE THV with a severe PPM, the treatment with an S-SE resulted in a better hemodynamic result.
• A no PPM after the first TAVR (HR 0.126, 0.31–0.51, *p* = 0.004) and a degenerated ≤ 23 mm THV (HR 19.7, 2.28–157.4, *p* = 0.006) are independent predictors of severe PPM after TAV-in-TAVR.
• At 1-year follow-up, the rates of all-cause mortality and the NYHA class III/IV were higher in the cohort of patients with severe PPM compared to those patients with moderate or no PPM.

Several studies investigated the incidence and clinical outcomes of PPM after surgical or transcatheter aortic valve replacement conveying conflicting results, mainly due to several methodological differences. Herrmann et al. (3) found that severe PPM was present in 12% of patients treated by means of TAVR and it was associated with a higher 1-year mortality, and heart failure (HF) rehospitalization. Okuno et al. (12) found that the rate of severe PPM was significantly lower in patients undergoing TAVR with a self-expanding device compared to those patients treated with a balloon-expandable device (6.7 vs. 15.6%; *p* = 0.003) with no impact of PPM on cardiovascular mortality or the NYHA class at 1 year. Recently, an analysis of the TVT Registry, including patients undergoing TAVR with self-expanding THVs, showed a rate of severe PPM of 5.3% in patients undergoing *de novo* TAVR and 27% in those patients undergoing TAVR-ViV (13). It is also well established that the results of TAVR-ViV for failed surgical bioprostheses are significantly conditioned by the presence of a preexisting severe PPM, an elevated postprocedural gradient, or a *de novo* mismatch (14). Strategies aiming to reduce the risk of a post-TAVR-ViV severe mismatch include high transcatheter valve implantation (0–2 mm below the prosthesis sewing ring), the use of a supra-annular self-expanding THV, and the use of techniques such as bioprosthetic valve fracture or remodeling (14, 15).

The incidence and clinical impact of the different grades of PPM are unknown in the field of TAV-in-TAVR.

In our study, including 155 patients with a degenerated THV treated by means of a second TAVR, the rates of severe and moderate PPM were 6.5 and 14.2%, respectively, thus slightly higher than that observed in published series on *de novo* TAVR, but lower as compared to TAVR-ViV (12). The latter might be explained by the larger EOA of the TAVR technologies: it is conceivable that, on average, a degenerated TAVR could have a larger EOA than a degenerated surgical bioprosthesis. This condition obviously allows the implantation of a relatively larger second THV.

Of note, we found a significantly higher rate of severe PPM in patients receiving a second S-SE platform into a degenerated BE THV (10.5%, *p* = 0.04): a possible explanation for this finding might be the fact that almost all the patients presenting with a severe PPM after the second TAVR actually had a degenerated THV ≤ 23 mm. In other words, in the presence of quite small anatomy, even the supra-annular position, which is associated with a larger EOA, might not be enough to resolve the PPM (Figure 1).

On the other hand, the finding that the use of a BE THV to treat a degenerated BE THV might imply a higher risk of at least a moderate PPM that might be explained by the double intra-annular position, which is surely related to an avoidable reduction of the orifice (Figure 1).

After the first TAVR, 8 patients had a severe PPM: all of them with a degenerated BE THV and 7 out of 8 patients with a ≤ 23 mm BE THV (see Supplementary Tables 1, 2). These patients have been treated in all the cases with an S-SE and, after the second TAVR, only in 1 case, there was still a severe PPM (Table 7). This might be explained by the significantly larger EOA of an S-SE THV, which seems to be a reasonable choice to treat a degenerated BE THV, in the absence of a significant risk of coronary obstruction/sinus sequestration.

A no PPM after the first TAVR is a negative predictor of a severe PPM, while a severe PPM after the first TAVR is a strong positive predictor. Considering the low number of cases with severe PPM, and the relatively small sample size, the multivariate analysis is of a pure hypothesis-generating nature; however, these results seem realistic.

Finally, consistently with the available literature (3, 12, 13), we found a significantly higher rate of 1-year all-cause mortality and the NYHA class III/IV in patients with severe PPM (*p* = 0.02 and *p* = 0.0001, respectively). Whether this can be completely ascribed to the presence of severe PPM or is influenced by increased frailty, presence of significant comorbidity and reduced functional status as reflected by the presence of significantly higher risk scores (Table 1) should be further evaluated.

## Clinical implications and avenues for future research

The techniques of bioprosthetic valve fracture/remodeling and BASILICA have been successfully applied to the field of ViV to reduce the risk of residual high gradient and coronary obstruction/sinus sequestration in patients with a degenerated surgical bioprosthesis (14, 15). Their role in the field of TAV-in-TAVR is completely unknown. However, the therapeutic strategy in the case of degeneration of the THV should probably be part of the routine evaluation done by the heart team, in particular when dealing with patients with long-life expectancy. In other words, it is quite realistic that the number of patients with a degenerated TAVR will tend to increase in the future.

Clearly, very fragile or old patients will unlikely experience a structural valve deterioration considering their inherent risk of mortality (4): in these cases, the selection of the most appropriate THV should only respect the criteria of feasibility and safety.

Our data also pointed out the importance of the anatomy and, as a consequence, of the choice of the first THV, at the beginning of the “valve journey”: small anatomy is obviously the real challenge for the reintervention, as it poses a high risk of coronary obstruction/flow impairment, as well as of severe PPM.

An S-SE might be associated with better durability (14), thus suggesting that it would be the first choice in patients with longer life expectancy; however, it is obvious that an S-SE with high commissure in small anatomy would be at extreme risk for coronary occlusion in case of a reintervention. On the other hand, a BE in small anatomy may be more prone to degenerate because of a higher chance of significant PPM (16); in this case, the treatment with an S-SE, provided suitable anatomy of the aortic root, seems to be promising.

Overall, a tailored approach at the time of the first TAVR is becoming critically important and the implementation of implantation techniques aiming at the commissure-to-commissure alignment should be pursued in every case in order to minimize the subsequent risk of coronary flow impairment and difficult coronary reaccess. Similarly, the evaluation of the risk of significant PPM, which is more likely with BE THVs, should be evaluated with the risk of PVL that, on the contrary, seems to favor the BE THVs, likewise the risk of pacemaker implantation (17–19).

## Limitations

Being an investigator-initiated registry, no central adjudication of events has been performed and echo data have been collected by the participating centers. The relatively low sample size does not allow definite conclusions, indeed the latter should be viewed as hypothesis-generating; however, this is the largest series in the field of TAV-in-TAVR and the present analyzes of the PPM may serve to generate and design future studies.

## Conclusion

The rate of moderate and severe PPM after TAV-in-TAVR is lower than that observed after TAVR-ViV, but, as expected, higher than TAVR in native aortic annuli. A severe PPM is associated with increased 1-year mortality and reduced functional capacity. At the time of the first treatment, a modern approach to TAVR should consider the possible future need for a reintervention and its implications, especially when evaluating patients with long-life expectancy in whom a structural valve deterioration is likely to occur.

## Impact on daily practice

- Following the degeneration of a THV, the procedure of TAV-in-TAVR will surely be progressively more frequent.

- After a TAV-in-TAVR, the risk of severe PPM is more frequent with specific first-second THVs combinations and it is significantly more frequent when a severe PPM was present yet after the first TAVR.

- A severe PPM implies a higher rate of both the 1-year mortality and the NYHA class III/IV, thus a careful evaluation should be made at the time of the first procedure, at the beginning of the “valve journey.”

## Data availability statement

The original contributions presented in this study are included in the article/Supplementary material, further inquiries can be directed to the corresponding author.

## Ethics statement

The studies involving human participants were reviewed and approved by the ECs of every participating center have been informed. The patients/participants provided their written informed consent to participate in this study.

## Author contributions

MC and EC drafted the manuscript. LT and FB provided expert revision. MC performed the statistical analysis. All authors contributed to the collection of the data and provided critical comments to the manuscript.
