# Supplementary material for: Prosthesis-patient mismatch following transcatheter aortic valve replacement for degenerated transcatheter aortic valves: the TRANSIT-PPM international project
Source: Front Cardiovasc Med. 2022 Jul 29;9:931207. doi: 10.3389/fcvm.2022.931207 (PMC9372302; doi:10.3389/fcvm.2022.931207)
Supplement: Supplementary file 1 [file Data_Sheet_1.pdf]

SUPPLEMENTARY TABLES

S1. Baseline PPM according to the type of THV (see text for the acronyms)

	OVERALL POPULATION (N=155)	S-SE (N=73)	BE (N=82)	P VALUE
SEVERE PPM	8 (5.2%)	0 (0)	8 (13.8%)	0.02
MODERATE PPM	32 (20.6%)	9 (15.7%)	23 (39%)	0.008
NO PPM	115 (74.2%)	64 (84.3%)	50 (47.2%)	0.0001

S2. PPM after 1st TAVR, according to the size ≤ 23 or >23

	OVERALL POPULATION (N=155)	1ST TAVR ≤ 23 MM (N=55)	1ST TAVR > 23 MM (N=100)	P VALUE
SEVERE PPM	8 (5.2%)	7 (12.7%)	1 (1%)	0.003
MODERATE PPM	32 (20.6%)	21 (38.2%)	11 (11%)	0.0001
NO PPM	115 (74.2%)	27 (49.1%)	88 (88%)	0.0001

S3. Iterations of 1st and 2nd THV according to the size of the first THV

	OVERALL (N=155)	SUPRANNULAR IN SUPRANNULAR (N=51)	SUPRANNULAR IN INTRANNULAR (N=36)	INTRANNULAR IN SUPRANNULAR (N=22)	INTRANNULAR IN INTRANNULAR (N=46)	P
1ST TAVR ≤ 23 MM	55 (35.5%)	4 (7.8%)	27 (75%)	0 (0)	24 (52.2%)	0.002
1ST TAVR > 23 MM	100 (64.5%)	47 (92.2%)	9 (25%)	22 (100%)	22 (47.8%)	0.0001
